# Deepath-MSI: a clinic-ready deep learning model for microsatellite instability detection in colorectal cancer using whole-slide imaging

**DOI:** 10.1038/s41698-025-01094-2

**Published:** 2025-08-28

**Authors:** Xu Feng, Wenjuan Yin, Qing Ye, Yayun Chi, Huer Wen, Yifeng Sun, Jin Zheng, Qifeng Wang, Qian Wang, Ming Zhao, Lin Yuan, Qinghua Xu, Dan Su, Xiaoyan Zhou

**Affiliations:** 1 Department of Pathology, Fudan University Shanghai Cancer Center, Shanghai, China; 2 Department of Oncology, Shanghai Medical College, Fudan University, Shanghai, China; 3 Institute of Pathology, Fudan University, Shanghai, China; 4 Department of Pathology, Zhejiang Cancer Hospital, Hangzhou, Zhejiang China; 5 Hangzhou Institute of Medicine (HIM), Chinese Academy of Sciences, Hangzhou, Zhejiang China; 6 Department of Pathology, The First Affiliated Hospital of University of Science and Technology of China (USTC), Division of Life Sciences and Medicine, University of Science and Technology of China, Hefei, China; 7 Department of Pathology, Shanghai General Hospital, Shanghai Jiaotong University School of Medicine, Shanghai, China; 8 Canhelp Genomics Research Center, Canhelp Genomics Co., Ltd., Hangzhou, China; 9 College of Computer Science and Technology, Tongji University, Shanghai, China; 10 Shanghai Key Laboratory of Medical Epigenetics, Institutes of Biomedical Sciences, Fudan University, Shanghai, China; 11 Ningbo Clinical Pathology Diagnosis Center, Ningbo, China

**Keywords:** Cancer imaging, Gastrointestinal cancer, Tumour biomarkers

## Abstract

Microsatellite instability (MSI) is crucial for immunotherapy selection and Lynch syndrome diagnosis in colorectal cancer. Despite recent advances in deep learning algorithms using whole-slide images, achieving clinically acceptable specificity remains challenging. In this large-scale multicenter study, we developed Deepath-MSI, a feature-based multiple instances learning model specifically designed for sensitive and specific MSI prediction, using 5070 whole-slide images from seven diverse cohorts. Deepath-MSI achieved an AUROC of 0.98 in the test set. At a predetermined sensitivity threshold of 95%, the model demonstrated 92% specificity and 92% overall accuracy. In a real-world validation cohort, performance remained consistent with 95% sensitivity and 91% specificity. Deepath-MSI could transform clinical practice by serving as an effective pre-screening tool, substantially reducing the need for costly and labor-intensive molecular testing while maintaining high sensitivity for detecting MSI-positive cases. Implementation could streamline diagnostic workflows, reduce healthcare costs, and improve treatment decision timelines.

## Introduction

Colorectal cancer (CRC) is the third most commonly diagnosed cancer globally and the second most prevalent malignancy in China^1,2^. Microsatellite instability (MSI), resulting from defects in the DNA mismatch repair (MMR) system, occurs in approximately 15% of CRC cases in Western populations and 7.4% in Chinese populations^3,4^. MSI status is crucial in informing immunotherapy choices, assessing patient prognosis, and diagnosing Lynch syndrome^5^.

Immunohistochemistry (IHC) and polymerase chain reaction (PCR) are the most commonly used methods for detecting MSI, as recommended by clinical guidelines^6,7^. However, both approaches demand experienced pathologists and specialized technicians to conduct the tests and interpret the outcomes, making them resource- and time-intensive, especially in low-resource settings.

Deep learning-based artificial intelligence (AI) has emerged as a transformative tool for biomarker prediction using routine hematoxylin and eosin (H&E) stained slides^8^. Omar et al. describes a practical workflow for solid tumor associative modeling in pathology (STAMP), enabling prediction of biomarkers directly from whole-slide images (WSIs) by using deep learning^9^. Deep learning models developed from WSIs of tumor H&E slides have demonstrated significant potential in predicting MSI status in CRC, with area under the receiver operating characteristic curve (AUROC) values reported between 0.85 and 0.96 across several studies^10–13^. Notably, MSIntuit–the first CE-IVD-approved AI-based product achieved high sensitivity (96–98%) but modest specificity (46–47%), underscoring AI’s ability to rule out MSI-negative cases while highlighting the need for specificity improvements^14^.

To address this challenge, we developed Deepath-MSI, a feature-based multiple instance learning model trained on 5070 primary colorectal tumor WSIs from seven geographically diverse, independent cohorts. Model performance was rigorously assessed using both an independent multicenter test set and a real-world validation cohort to establish clinical utility. Deepath-MSI demonstrated excellent discrimination in the multicenter test set (AUROC = 0.98), achieving 92% specificity and 92% overall accuracy at a 95% sensitivity operating point. In the real-world cohort, Deepath-MSI maintained high sensitivity (94.6%) against standard methods (such as IHC and PCR) and correctly excluded 90.7% of non-MSI-H tumors. This robust performance led to Deepath-MSI receiving “Breakthrough Device” designation from China’s National Medical Products Administration (NMPA) on March 26, 2025, marking its approval as the AI-driven, deep-learning-based Class III Innovative Medical Device in digital pathology.

## Results

### Dataset description

**Fig. 1:**
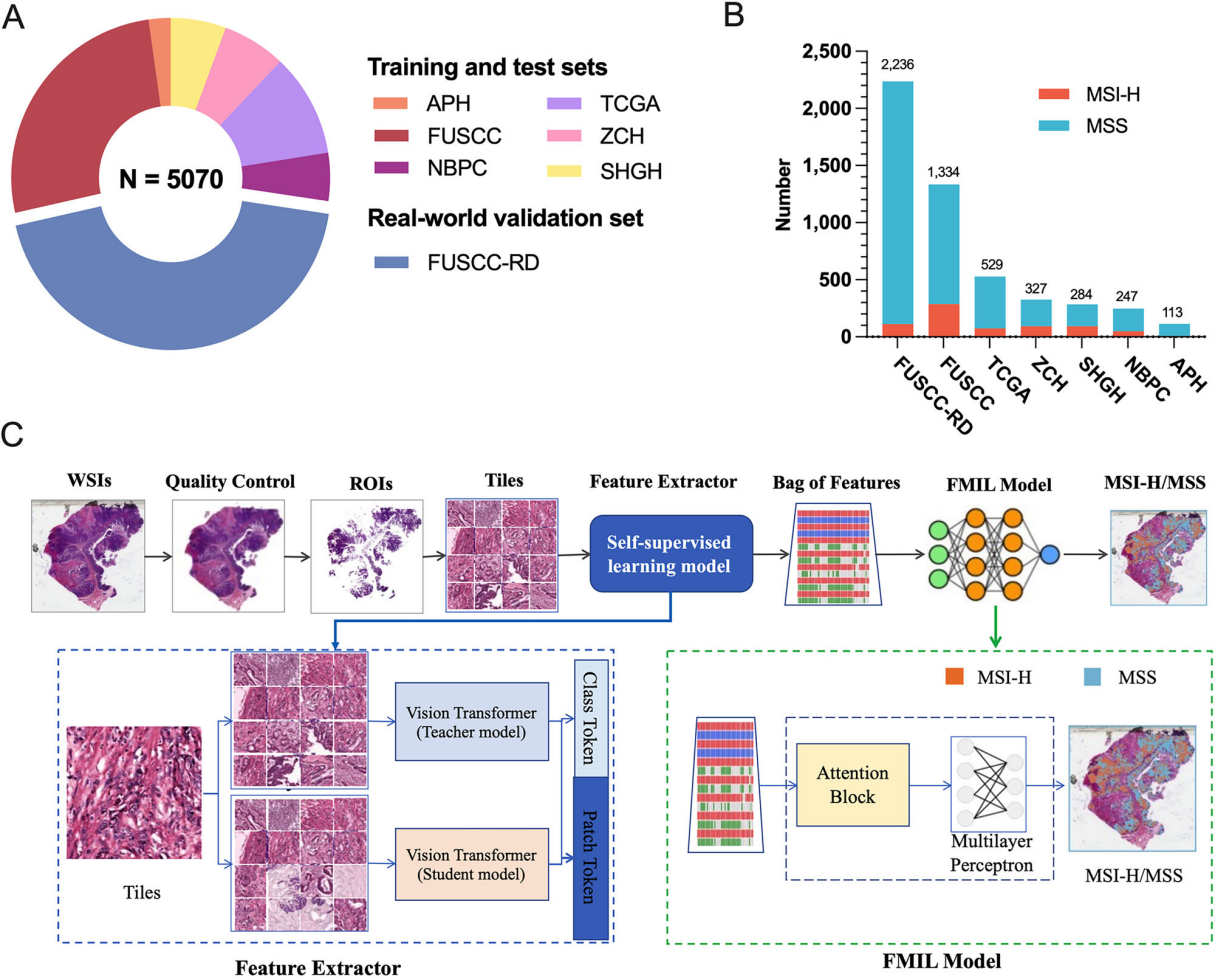
Cohort and workflow overview. **A** Overview of the seven cohorts of colorectal cancer (FUSCC-RD, *n* = 2236; FUSCC, *n* = 1334; TCGA, *n* = 529; ZCH, *n* = 327; SHGH, *n* = 284; NBPC, *n* = 247; APH, *n* = 113). **B** Distribution of cases across the seven cohorts. **C** Workflow overview of model architecture.

Among the total cases (*N* = 5070), 712 cases (14.0%) were identified as MSI-H/dMMR, while 4358 cases (86.0%) were classified as MSS/pMMR (Fig. 1A, Table S1). The distribution of cases across the seven cohorts is illustrated in Fig. 1B. WSIs from six cohorts (APH, FUSCC, NBPC, SHGH, ZCH, and TCGA) were randomly divided into a training set (*n* = 1600) and a test set (*n* = 1234). To further assess the model’s performance, we included an independent real-world validation set (FUSCC-RD), consisting of consecutively collected, surgically resected primary colorectal cancer specimens. In this cohort, MSI status was determined by IHC for MLH1, MSH2, MSH6, and PMS2, with cases classified as dMMR if any of the four proteins were deficient. Importantly, the FUSCC-RD cohort is completely non-overlapping with the FUSCC cohort, in which MSI status was assessed either PCR or NGS. Clinical characteristics of training and test sets are summarized in Table S2, and real-world validation set characteristics are provided in Table 2.

### Performance of the Deepath-MSI model across cohorts

**Fig. 2:**
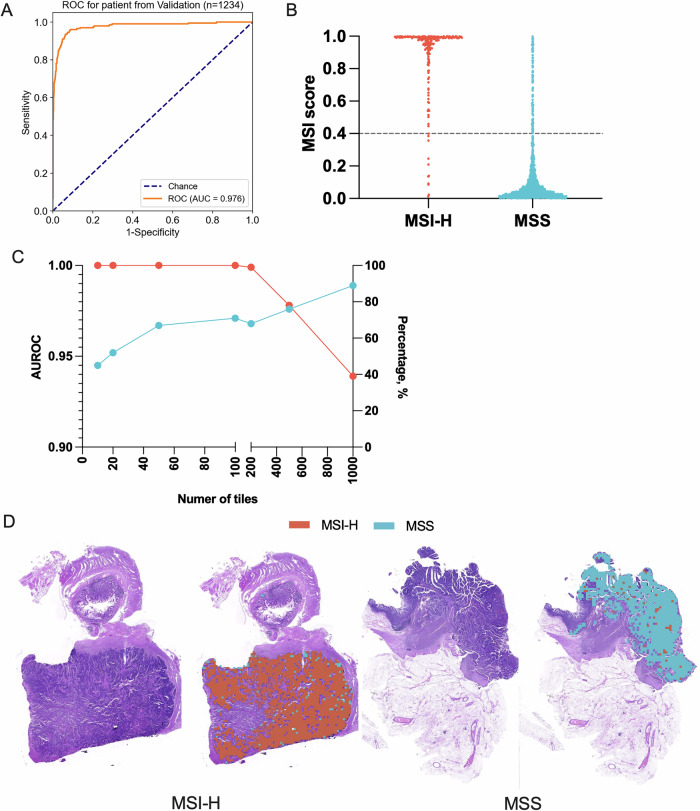
Performance evaluation of the model in the test set. **A** AUROC for the test set. **B** Distribution of MSI scores in MSI-H and MSS specimens. **C** Relationship between AUROC, the percentage of included specimens and the number of tiles per specimen. **D** Representative examples of MSI-H and MSS predictions.

In this study, a Deepath-MSI model was employed to predict MSI status directly from digitized H&E slides of CRC tissue (Fig. 1C). Our findings indicate that MSI status can be accurately determined using histopathological images, with the model achieving an AUROC of 0.976 in test set (Table 1, Fig. 2A). The Deepath-MSI model displayed robust performance across various cohorts. Specifically, AUROC values ranged from 0.967 in the NBPC cohort to 0.988 in the SHGH cohort. Scanner variability was also assessed, with AUROC values ranging from 0.950 for the 3D Histech scanner to 0.991 for the KFBIO scanner. Population-based analyses further revealed that the model performed similarly in the Chinese population (AUROC 0.977) compared to the Western population (AUROC 0.968) (Figs. S1–2).

**Table 1:** Performance of the Deepath-MSI model

	Sample Size	MSI-H	MSS	Positive Ratio	AUROC	Threshold	Sensitivity	Specificity	Accuracy	PPV	NPV
**Dataset - Overall**											
Training Set	1600	400	1200	25.00%	0.996	0.4	98.00%	95.80%	96.30%	88.49%	99.31%
Test Set	1234	201	1033	13.30%	0.976	0.4	95.00%	91.70%	92.20%	68.95%	98.96%
**Test Set – Individual Centers**											
APH	49	3	46	6.10%	0.986	0.4	100.00%	95.70%	95.90%	60.00%	100.00%
FUSCC	545	84	461	15.40%	0.972	0.4	92.90%	90.20%	90.60%	63.41%	98.58%
NBPC	102	14	88	13.70%	0.967	0.4	100.00%	90.90%	92.20%	63.64%	100.00%
TCGA	223	22	201	9.90%	0.968	0.4	90.90%	93.50%	93.30%	60.61%	98.95%
ZCH	131	27	104	20.60%	0.978	0.4	96.30%	92.30%	93.10%	76.47%	98.97%
SHGH	184	51	133	27.70%	0.988	0.4	98.00%	92.50%	94.00%	83.33%	99.19%
**Digital Scanner**											
3D Histech	319	50	269	15.70%	0.95	0.4	88.00%	91.10%	90.60%	64.71%	97.61%
KFBIO	541	112	429	20.70%	0.991	0.4	98.20%	90.90%	92.40%	73.83%	99.49%
Leica	223	22	201	9.90%	0.968	0.4	90.90%	93.50%	93.30%	60.61%	98.95%
Shengqiang	151	17	134	11.30%	0.971	0.4	100.00%	92.50%	93.40%	62.96%	100.00%
**Population**											
Chinese	1011	179	832	17.70%	0.977	0.4	95.50%	91.20%	92.00%	70.08%	98.96%
Western	223	22	201	9.90%	0.968	0.4	90.90%	93.50%	93.30%	60.61%	98.95%

To enhance clinical utility, we established an optimal MSI score threshold for the Deepath-MSI model by fixing the sensitivity at 95% across the test set. This yielded an MSI score threshold of 0.4, at which the model achieved a sensitivity of 95.0%, specificity of 91.7%, PPV of 69.0%, NPV of 99.0% and an overall accuracy of 92.2% (Table 1).

The distribution of MSI score in test set was shown in Fig. 2B. To ensure sufficient representation of tumor tissue and model reliability, we determined the minimum number of tumor tiles required for quality control. From the analysis of the test set, we established a requirement of at least 100 tiles, achieving an AUROC of 0.971 and covering 100% of samples (Fig. 2C, Table S3). This threshold corresponds to approximately 6.6 mm² of tumor area, ensuring stable predictive performance. Figure 2D represents representative examples of MSI-H and MSS predictions.

### Diagnostic performance on real-world validation set

**Fig. 3:**
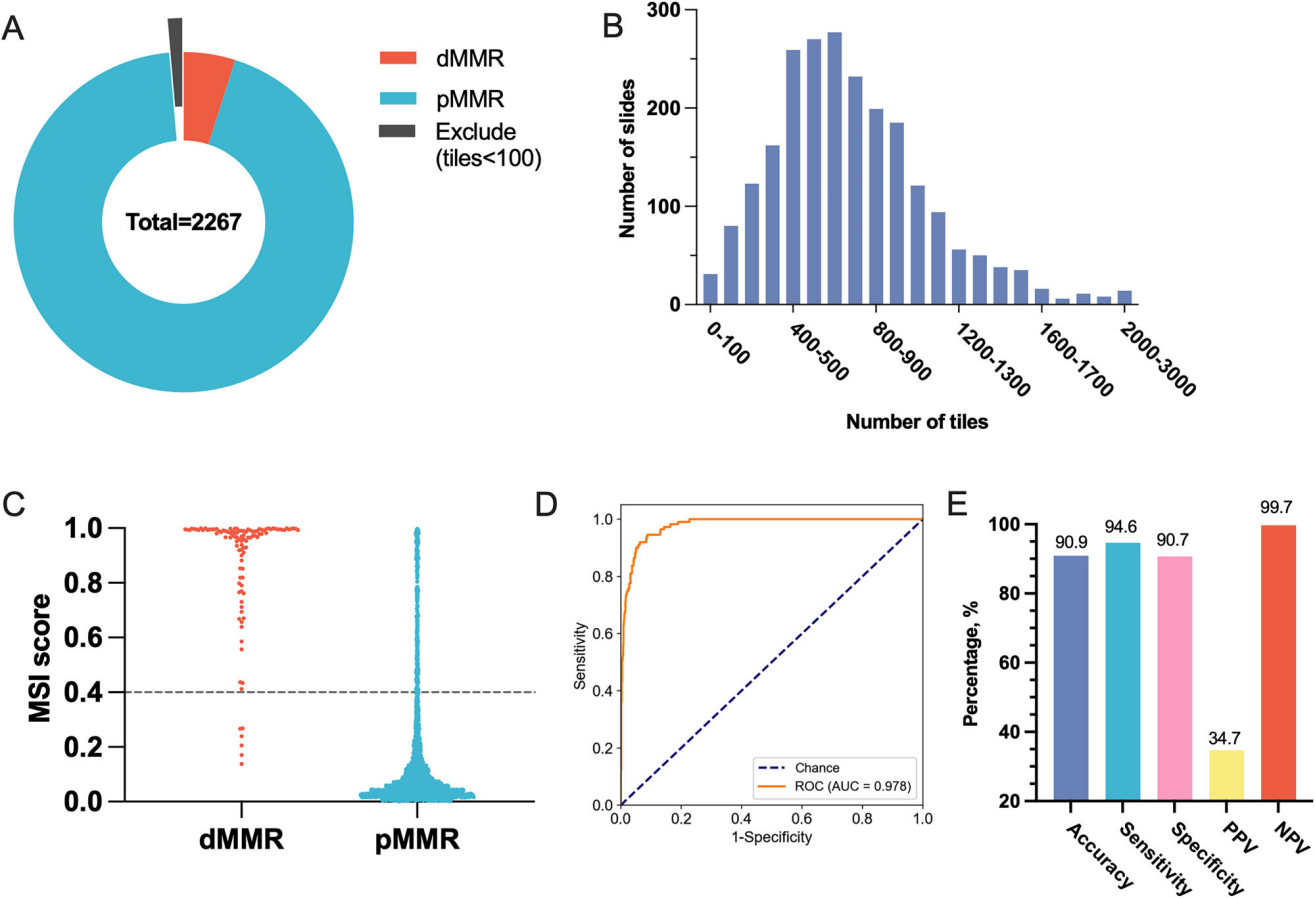
Clinical workflow. Performance evaluation of the model in the real-world validation set. **A** Overview of the real-world validation set with dMMR/pMMR status. **B** Distribution of the number of tiles per slide for real-world validation set. **C** Distribution of MSI scores in dMMR and pMMR specimens. **D** AUROC for the real-world validation set. **E** The overall accuracy, sensitivity, specificity, PPV and NPV of the model in the real-world validation set.

Initially, 2267 colorectal cancer cases were included. Of these, 2236 cases meet the minimum tumor tiles (*n* = 100) and enrolled in this study, achieving a successful rate of 98.6% (2236/2267) (Fig. 3A). Among the enrolled cases, 111 (5.0%) were classified as dMMR, while the remaining 2125 (95.0%) were classified as pMMR. Detailed clinicopathological features are presented in Table 2. The dMMR ratio showed a significant association with factors such as age, primary tumor site, tumor size, histological type and tumor differentiation.

**Table 2:** Clinicopathological features and performance of Deepath-MSI model in the real-world validation set

	Number	dMMR	pMMR	dMMR ratio	*p*	Accuracy	*p*	Sensitivity	*p*	Specificity	*p*	PPV	*p*	NPV	*p*
**Overall**	2236	111	2125	5.00%		90.90%		94.60%		90.70%		34.70%		99.70%	
**Gender**															
Male	1376	59	1317	4.30%	0.071	90.80%	1.000	91.50%	0.212	90.80%	0.818	30.90%	0.113	99.60%	0.418
Female	860	52	808	6.00%		90.90%		98.10%		90.50%		39.80%		99.90%	
**Age**															
Median (IQR)	62 (54, 69)														
<50	298	27	271	9.10%	0.001	89.30%	0.330	96.30%	1.000	88.60%	0.218	45.60%	0.064	99.60%	0.551
≥50	1938	84	1854	4.30%		91.10%		94.00%		91.00%		32.10%		99.70%	
**Primary tumor site**															
Rectum	969	13	956	1.30%	<0.001	94.00%	<0.001	76.90%	0.033	94.20%	<0.001	15.40%	0.002	99.70%	0.945
Sigmoid colon	350	9	341	2.60%		92.90%		88.90%		93.00%		25.00%		99.70%	
Ascending colon	321	43	278	13.40%		82.60%		97.70%		80.20%		43.30%		99.60%	
Rectosignmoid colon	202	5	197	2.50%		93.10%		80.00%		93.40%		23.50%		99.50%	
Descending colon	106	8	98	7.50%		91.50%		100.00%		90.80%		47.10%		100.00%	
Transverse colon	103	15	88	14.60%		83.50%		100.00%		80.70%		46.90%		100.00%	
Others	185	18	167	9.70%		86.50%		100.00%		85.00%		41.90%		100.00%	
**Primary tumor site (Left/Right)**^**a**^															
Left hemicolon	730	28	702	3.80%	<0.001	92.70%	<0.001	92.90%	0.196	92.70%	<0.001	33.80%	0.159	99.70%	1.000
Right hemicolon	518	70	448	13.50%		82.60%		98.60%		80.10%		43.70%		99.70%	
**Tumor size (cm)**^**b**^															
<3	463	15	448	3.20%	<0.001	92.90%	<0.001	100.00%	0.238	92.60%	<0.001	31.30%	0.105	100.00%	0.339
3 ~ 6	1450	51	1399	3.50%		92.30%		90.20%		92.40%		30.30%		99.60%	
>6	311	45	266	14.50%		81.00%		97.80%		78.20%		43.10%		99.50%	
**Histological type**															
Adenocarcinoma	2021	86	1935	4.30%	<0.001	92.20%	<0.001	94.20%	0.573	92.10%	<0.001	34.60%	0.205	99.70%	0.239
Adenocarcinoma with mucus	94	14	80	14.90%		85.10%		100.00%		82.50%		50.00%		100.00%	
Mucinous adenocarcinoma	72	9	63	12.50%		65.30%		88.90%		61.90%		25.00%		97.50%	
Others	49	2	47	4.10%		85.70%		100.00%		85.10%		22.20%		100.00%	
**Tumor differentiation**^**c**^															
Well	33	2	31	6.10%	<0.001	84.80%	<0.001	100.00%	0.065	83.90%	<0.001	28.60%	0.201	100.00%	0.155
Well-moderate	104	3	101	2.90%		91.30%		66.70%		92.10%		20.00%		98.90%	
Moderate	1273	45	1228	3.50%		92.50%		91.10%		92.50%		30.80%		99.60%	
Moderate-poorly	622	43	579	6.90%		91.30%		100.00%		90.70%		44.30%		100.00%	
Poorly	177	17	160	9.60%		79.70%		94.10%		78.10%		31.40%		99.20%	
**Number of tiles**															
100 ~ 200	80	4	76	5.00%	0.027	92.50%	0.316	50.00%	0.011	94.70%	0.107	33.30%	0.551	97.30%	0.033
200 ~ 500	544	17	527	3.10%		92.30%		94.10%		92.20%		28.10%		99.80%	
500 ~ 1000	1163	57	1106	4.90%		90.90%		94.70%		90.70%		34.40%		99.70%	
>1000	449	33	416	7.30%		88.90%		100.00%		88.00%		39.80%		100.00%	

^a^A total of 1248 samples were derived from left- or right- hemicolon tumors, ^b^Tumor size data were missing for 12 cases, ^c^Tumor differentiation data were missing for 27 cases.

In the real-world validation set, the median number of tiles per sample was 681, with a range from 106 to 2924 (Fig. 3B). The distribution of MSI score in real-world validation set was shown in Fig. 3C. With a prespecified threshold of 0.4, the model achieved AUROC of 0.978 (Fig. 3D), along with a sensitivity of 94.6%, a specificity of 90.7%, a PPV of 34.7%, a NPV of 99.7% (Fig. 3E). Overall, 90.9% of specimens were accurately classified according to their true MSI status. Additionally, performance was evaluated across various clinicopathological subgroups. Notably, the model exhibited relatively diminished performance in specific subgroups, including those with tumors located in the ascending or transverse colon, right-sided colon cancer, tumors larger than 6 cm, tumors present with mucus, or poorly differentiated tumors (Table 2).

## Discussion

Recent advancements in predicting MSI status from WSIs have been promising, with numerous studies reporting high AUROCs of 0.85 to 0.96^10–13^. A commonly utilized method involves fully supervised approaches that assume a uniform MSI status across each WSI, applying a consistent slide-level label to all constituent tiles during training^15^. However, this approach overlooks the heterogeneity in the distribution of MSI-H and MSS patterns within a WSI, which may impact the performance of models. To address this limitation, weakly supervised learning methods, particularly MIL, have shown significant advantage by enabling more accurate MSI status prediction based on only slide-level labels. In our study, Deepath-MSI model demonstrated superior performance over previous weakly supervised model in surgical samples, achieving an AUROC of 0.976 compared to 0.84^16^.

Interestingly, while some models have shown impressive performance, two studies on TCGA cohorts observed notably lower AUROCs. For instance, MSINet achieved an AUROC of 0.779, whereas WiseMSI’s performance was comparatively lower, with an AUROC of 0.632^12,13^. In our study, the Deepath-MSI model demonstrated superior performance relative to these prior models. Moreover, when comparing Deepath-MSI with MSIntuit, the sensitivities of the two models were comparable, with Deepath-MSI and MSIntuit both achieving sensitivities of approximately 95%. However, Deepath-MSI demonstrated markedly higher specificity at 90.7–91.7%, compared to MSIntuit’s 46–47%, potentially excluding up to 44% more MSS CRC patients from unnecessary further molecular testing^14^.

The pathological characteristics observed in dMMR cases within the FUSCC-RD cohort were largely consistent with previously reported clinicopathological patterns of dMMR colorectal cancers. Specifically, dMMR tumors in our cohort were more frequently located in the right-sided colon, exhibited larger size (>6 cm), poor differentiation, and mucinous histology, and were more common in younger patients. These features have been well-documented in multiple studies as hallmarks of MSI-H colorectal cancers, reflecting distinct tumor biology and clinical behavior^3,17^. The alignment between our real-world data and established literature underscores the clinical validity and generalizability of our findings, further supporting the utility of our dataset for evaluating the diagnostic performance of Deepath-MSI in routine clinical practice. To evaluate Deepath-MSI model’s performance across clinicopathological subgroups, we identified patterns of false MSI predictions. Similar to findings reported by Wu and Echle^16,18^, false predictions in our model were more frequent in cases involving tumors located in the right-sided colon, and tumors present with mucus. Additionally, our analysis indicated that false predictions were also associated with tumors larger than 6 cm or those with poor differentiation. These findings underscore the potential influence of clinicopathological characteristics on MSI prediction accuracy and highlight specific patient subgroups that may benefit from model refinements to improve predictive reliability.

Traditional AI model relied on supervised deep learning, necessitating expert pathologist annotations for each pathological image, a process that is both time-consuming and resource-intensive^19^. To overcome these challenges, this study introduces a novel preprocessing step for digitized pathological images, enabling efficient identification of ROI and significantly minimizing the need for extensive pathologist annotation. A key challenge in implementing AI systems in clinical practice is their limited generalizability, often attributed to variations in the color and brightness of H&E-stained slides across medical centers, scanner-related technical differences, and histological diversity between populations^20^. To address this, our training set incorporated a robust, multi-center dataset, comprising both Western and Chinese populations across five institutions. In addition, the feature extractor was trained on a large number of tiles with extensive data augmentation, without the use of any labels. The diverse data collection and feature extractor strengthened the model’s ability to generalize across different ethnic and clinical environments. Our validation studies demonstrated that Deepath-MSI model maintained high performance not only across different scanners but also among various demographic groups and clinical centers.

**Fig. 4:**
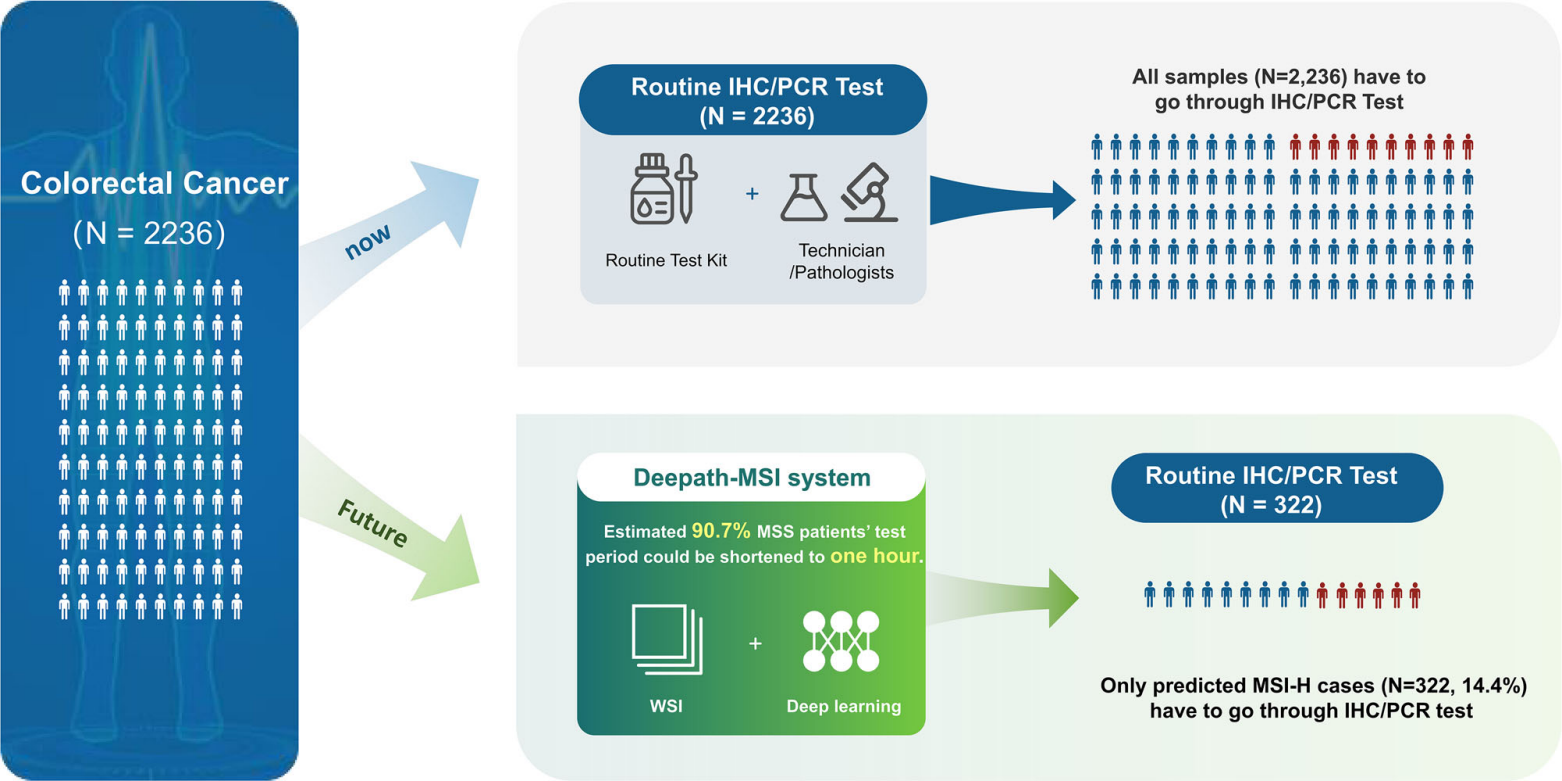
Integration of Deepath-MSI into the clinical workflow for efficient MSI screening. The diagram compares the current workflow, where all colorectal cancer cases (*N* = 2236) undergo IHC/PCR testing, with the proposed Deepath-MSI workflow. The proposed approach utilizes Deepath-MSI analysis of H&E slides for initial screening, directing only predicted MSI-H cases (*N* = 322, 14.4%) to confirmatory IHC/PCR. This potentially excludes approximately 90.7% of MSS patients from further testing, significantly reducing the burden of ancillary tests.

Traditionally, CRC patients undergo testing for MSI/MMR status, a process that will take several days or even weeks using IHC or PCR methods^14^. Pathology laboratories are under increasing pressure to meet the rising demand for biomarker testing, exacerbated by a global shortage of pathologists. In low- and middle-income countries, this testing may be further hindered by resource limitations. AI-based approaches offer the advantage of rapid, automated analysis, easing the burden on pathologists and streamlining clinical workflows, particularly in high-throughput or resource-constrained settings. In a future real-world context (Fig. 4), the diagnosed H&E slides of CRC patients would first be assessed with the Deepath-MSI model within one hour, potentially allowing those classified as MSS to bypass routine IHC or PCR testing. Only patients predicted to be MSI-H would require confirmatory testing. This approach could reduce the testing workload by 85.6% (1914/2236), significantly shortening the time from surgery to molecular determination of MSI status, and enabling earlier initiation of immunotherapy, when indicated. Its robust performance across various clinical contexts highlights its potential for widespread adoption, especially in regions with limited access to advanced molecular diagnostic tools.

Although the Deepath-MSI model shows promise for predicting MSI status from digitized pathology images, several limitations warrant consideration. First, biopsy samples were not included in this study. Echle et al. report a lower performance on biopsy tissue than on surgical resection tissue (biopsy AUROC: 0.78, resection AUROC of 0.96)^10^. Wu et al. also find a significant performance gap between biopsy and surgical tissues, with lower AUROC in biopsy cohort (0.77 and 0.84, respectively)^16^. Ongoing validation studies are aimed at evaluating the Deepath-MSI model on biopsy samples to determine its utility in these contexts. Second, while the model achieved high accuracy within Chinese populations, its generalizability to other populations remains to be validated. Variations in tumor biology and histopathological features across populations necessitate large-scale studies to confirm its robustness across diverse ethnic and clinical settings.

In conclusion, this study develops a novel deep learning-based approach for pre-screening MSI using WSIs, offering a promising solution for accurate and efficient MSI evaluation in clinical practice. Future work will focus on prospectively multi-center validation of the Deepath-MSI model to further evaluate the model’s generalizability of this model. As artificial intelligence tools continue to advance in pathology, this approach has the potential not only to streamline the diagnostic process but also to improve patient outcomes by facilitating timely and appropriate therapeutic interventions.

## Methods

### Cohort description

In this multicenter retrospective study, we collected H&E-stained slides from formalin-fixed paraffin-embedded tissue sample obtained from seven independent CRC patient cohorts. Six of seven cohorts were derived from five Chinese institutions, including Anhui Provincial Hospital (APH), Fudan University Shanghai Cancer Center (FUSCC), Fudan University Shanghai Cancer Center-Real World (FUSCC-RD), Ningbo Pathology Center (NBPC), Shanghai General Hospital (SHPH) and Zhejiang Cancer Hospital (ZCH), with samples collected between January 2017 and February 2024. These cohort predominantly represent Chinese population. Ethics approval was granted by the ethics committee of the lead institution, FUSCC (Approval No. 2306276-4) and this study adhered to the ethical principles outlined in the Declaration of Helsinki. All patients provided written informed consent. The seventh cohort was derived from the Cancer Genome Atlas (TCGA) dataset, representing a Western population. MSI status for these cases was determined by integrating clinical information retrieved from cBioPortal (https://www.cbioportal.org/) with corresponding pathology reports downloaded from the Genomic Data Commons (GDC) portal (https://portal.gdc.cancer.gov/). The inclusion of both Chinese and Western cohorts ensures multi-ethnic representation, enhancing the generalizability and clinical applicability of the Deepath-MSI model across diverse populations. In total, 5070 CRC cases were included in the study. A detailed description of datasets is provided in Table S1. The WSI formats varied across datasets, comprising SQDC (Shengqiang, Shenzhen, China), KFB (KFBIO, Ningbo, China), MRXS (3D Histech, Budapest, Hungary), and SVS (Leica, Wetzlar, Germany).

### Identification of region of interest

A novel preprocessing method was utilized to identify regions of interest (ROIs) as follows: first, WSIs were divided into smaller tiles, each measuring 256 × 256 μm (resolution: 32 μm/pixel). Second, tiles with more than 50% background (white area) or containing artifacts, such as blurry regions and pen markings, were excluded. To efficiently identify artefacts, we developed a robust two-stage algorithm that first applies pixel clustering to group similar regions in low-resolution tiles, followed by a deep learning model to accurately detect artifacts. Third, the nuclear-cytoplasmic ratio was employed as a key indicator to filter tiles as ROIs. Finally, corresponding high-resolution tiles (resolution: 0.5 μm/pixel) were extracted, and MSI status was annotated at the patient level. MSI status for each sample was determined using IHC, PCR, or next-generation sequencing (NGS). Based on these results, cases were classified by a pathologist as either microsatellite instability-high/deficient mismatch repair (MSI-H/dMMR) or microsatellite stable/proficient mismatch repair (MSS/pMMR).

### Feature extractor

To predict MSI status, we developed a Feature-based Multiple Instance Learning (FMIL) system, consisting of two primary components: a feature extractor and an aggregation module. The feature extractor is pretrained using an image self-supervised learning framework (DINOv2), a state-of-the-art self-supervised learning framework for image analysis^21^.

A known challenge in histopathological image analysis is the inconsistency in color and intensity among sections, even within the same institution. These inconsistencies arise from several factors, such as variations in tissue fixation affected by sample size, section thickness due to procedural differences, and the choice of staining reagents during H&E staining. Such variations can interfere with the efficacy of quantitative image analysis. To mitigate this issue, the feature extractor was trained on a large number of image tiles, utilizing extensive data augmentation techniques to simulate diverse conditions, and was trained in a completely label-free manner.

The feature extractor processes each image tile independently, transforming it into a compact embedding. Importantly, the feature extractor’s weights remain fixed during both the training and inference phases of the FMIL system, ensuring consistent feature extraction across all analyses.

### Self-learning pretraining

The self-supervised pretraining phase employs a student-teacher architecture, utilizing momentum-updated teacher weights. In this framework, the teacher network receives global crops of each image, while the student network processes both global and local crops with varying augmentation strengths.

Specifically, global crops are augmented with weaker augmentations, such as random resized cropping and horizontal flipping, while local crops undergo stronger augmentations, including color jittering, Gaussian blurring, and solarization. The difference in augmentation strength promotes learning more nuanced and localized features by the student model.

The total loss function is a weighted combination of the cross-entropy and iBOT losses:

1 $${L}_{total}={L}_{CE}+\lambda {L}_{iBOT}$$

where $\lambda$ balances the two losses.

Cross-Entropy Loss (*L*_*CE*_): This loss encourages local-to-global correspondence. It’s computed between the student’s output for local crops and the sharpened, centered output of the teacher for global crops of the same image:

2 $${L}_{CE}=-\mathop{\sum }\limits_{i=1}^{N}{P}_{t}^{globa{l}^{{\prime} }}(i)\log \,{P}_{s}^{local}(i)$$

${P}_{t}^{globa{l}^{{\prime} }}$ is the sharpened and centered teacher output for a global crop, and ${P}_{s}^{local}$ is the student output for a local crop. Centering and sharpening are applied to the teacher output:

3 $$C=\frac{1}{T}\mathop{\sum }\limits_{{\rm{t}}=1}^{T}{P}_{t}^{global}$$

4 $${P}_{t}^{globa{l}^{{\prime} }}=\frac{{P}_{t}^{global}-C}{\tau }$$

iBOT Loss (*L*_*iBOT*_): This loss promotes invariance to masking and encourages learning robust, context-aware representations. It’s calculated between the teacher’s output for a global crop and the student’s output for a masked global crop of the same image:

5 $${L}_{iBOT}=-\mathop{\sum }\limits_{{\rm{i}}=1}^{B}\log \frac{\exp (sim({f}_{i}^{t,global},{f}_{i}^{s,maskedglobal})/{\tau }^{{\prime} })}{{\sum }_{j=1}^{B}\exp (sim({f}_{i}^{t,global},{f}_{i}^{s,maskedglobal})/{\tau }^{{\prime} })}$$

Where ${f}_{i}^{t,global}$ is the teacher output for the global crop of image i, and ${f}_{i}^{s,maskedglobal}$ is the student output for the masked global crop of image i. $sim(\cdot ,\cdot )$ denotes cosine similarity, and ${\tau }^{{\prime} }$ is a temperature parameter. The pretrained encoder resulting from minimizing this combined loss is then used to initialize the encoder of our segmentation network.

### MSI classifier

To construct the MSI classifier, a MIL approach was employed, following methods previously established for handling WSI data^22,23^. Unlike approaches that rely on sub-sampling a fixed number of tiles, MIL utilizes all tiles from a patient’s WSI as a “bag” without assuming every tile within the bag directly reflects the MSI status, which allows for greater resilience to intratumor heterogeneity. Furthermore, an attention mechanism was incorporated within the decoder, providing access to the full scope of encoded information. This mechanism assigns variable attention weights to different input regions, identifying the significance of each token and allocating priority for the generation of output tokens at every step.

### Implementation

The Deepath-MSI model was implemented using PyTorch and trained on an NVIDIA RTX 4090 GPU with 24 GB of memory^24^. To address the class imbalance in the patient labels during training, tiles from the more abundant class were randomly under-sampled, thereby ensuring equal numbers of tiles from positive and negative classes. This approach facilitated the training of the Deepath-MSI model on tile-level-balanced datasets. For model deployment on the test partition during cross-validation or on the external validation set, no class balancing procedure was applied.

### Statistical analysis

Statistical endpoints included the AUROC, sensitivity, specificity, positive predictive value (PPV), and negative predictive value (NPV). The optimal MSI score threshold for the Deepath-MSI model was determined by fixing the sensitivity at 95% in the test set. To assess the minimum tumor tiles required per slide for optimal Deepath-MSI model performance and to establish quality control criteria for the real-world validation set, we randomly selected a specific number of tiles per slide in the test set, analyzing predictions only for slides meeting the minimum tile threshold. The MSI score threshold and minimum tile number were then applied to the real-world validation set. The association between clinicopathological features and performance was analyzed using Fisher’s exact test.

## Supplementary information

Supplementary material

## Data Availability

High-resolution diagnostic whole slide image data from TCGA, as well as the associated diagnoses, can be publicly accessed via the National Institutes of Health Genomic Data Commons. To protect patient privacy, pathology image datasets and other patient-related information of the collected in-house datasets are not publicly available. However, all these data can be accessed upon reasonable request by contacting the corresponding author via email.

## Code availability

The underlying code for this study is not publicly available but may be made available to qualified researchers on reasonable request from the corresponding author.
