# Supplementary material for: Deepath-MSI: a clinic-ready deep learning model for microsatellite instability detection in colorectal cancer using whole-slide imaging
Source: NPJ Precis Oncol. 2025 Aug 28;9:302. doi: 10.1038/s41698-025-01094-2 (PMC12394642; doi:10.1038/s41698-025-01094-2)

SUPPLEMENT

Contents

Table S1. Patient cohorts used in this study 2

Table S2. Clinical characteristics of training and test sets 3

Table S3. Impact of amount of tumor tiles on the Deepath-MSI model 5

Figure S1. Performance of the Deepath-MSI model in (A-F) centers, (G-J) scanners, and (K-L) race. 6

Figure S2. Confusion matrix of the Deepath-MSI model in (A-F) centers, (G-J) scanners, and (K-L) race. 7

Table S1. Patient cohorts used in this study

Cohort	Number	MSI-H/dMMR	MSS/pMMR	Positive ratio
**APH**	113	8	105	7.1%
**FUSCC**	1,334	287	1,047	21.5%
**FUSCC-RD**	2,236	111	2,125	5.0%
**NBPC**	247	48	199	19.4%
**TCGA**	529	74	455	14.0%
**ZCH**	327	92	235	28.1%
**SHGH**	284	92	192	32.4%
**Total**	5,070	712	4,358	14.0%

* APH, Anhui Provincial Hospital; FUSCC, Fudan University Shanghai Cancer Center; FUSCC-RD, Fudan University Shanghai Cancer Center-Real World; NBPC, Ningbo Pathology Center; SHGH, Shanghai General Hospital; ZCH, Zhejiang Cancer Hospital.

Table S2. Clinical characteristics of training and test sets

	**Training Set**	**Test Set**
**Number**	1,600	1,234
**Sex**		
**Male**	802	656
**Female**	588	425
**Unspecified**	210	153
**Age**		
**Median (range)**	62 (14-92)	64 (24-90)
**MSI-status**		
**MSI-H/dMMR**	400	201
**MSS/pMMR**	1,200	1,033
**Primary cancer site**		
**Colon**	898	677
**Rectum**	376	325
**Others**	117	81
**Unspecified**	209	151
**Histology type**		
**Adenocarcinoma**	762	637
**Adenocarcinoma with mucus**	70	46
**Mucinous adenocarcinoma**	70	49
**Others**	14	17
**Unspecified**	684	485
**Tumor differentiation**		
**Well**	25	14
**Well-moderate**	27	29
**Moderate**	450	405

**Moderate-poorly**	226	157
**Poorly**	98	63
**Unspecified**	774	566

Table S3. Impact of amount of tumor tiles on the Deepath-MSI model

# of Tiles	MSI-H/dMMR	MSS/pMMR	Total	Positive ratio	AUROC	Threshold	Sensitivity	Specificity	Accuracy
**10**	201	1,033	1,234	16.3%	0.945	0.091	0.953	0.703	0.739
**20**	201	1,033	1,234	16.3%	0.952	0.115	0.953	0.770	0.796
**50**	201	1,033	1,234	16.3%	0.967	0.241	0.953	0.876	0.887
**100**	201	1,033	1,234	16.3%	0.971	0.405	0.953	0.919	0.924
**200**	198	1,018	1,216	16.3%	0.968	0.261	0.952	0.889	0.898
**500**	173	786	959	18.0%	0.976	0.439	0.951	0.930	0.933
**1,000**	104	375	479	21.7%	0.989	0.545	0.963	0.944	0.947

Figure S1. Performance of the Deepath-MSI model in (A-F) centers, (G-J) scanners, and (K-L) race.

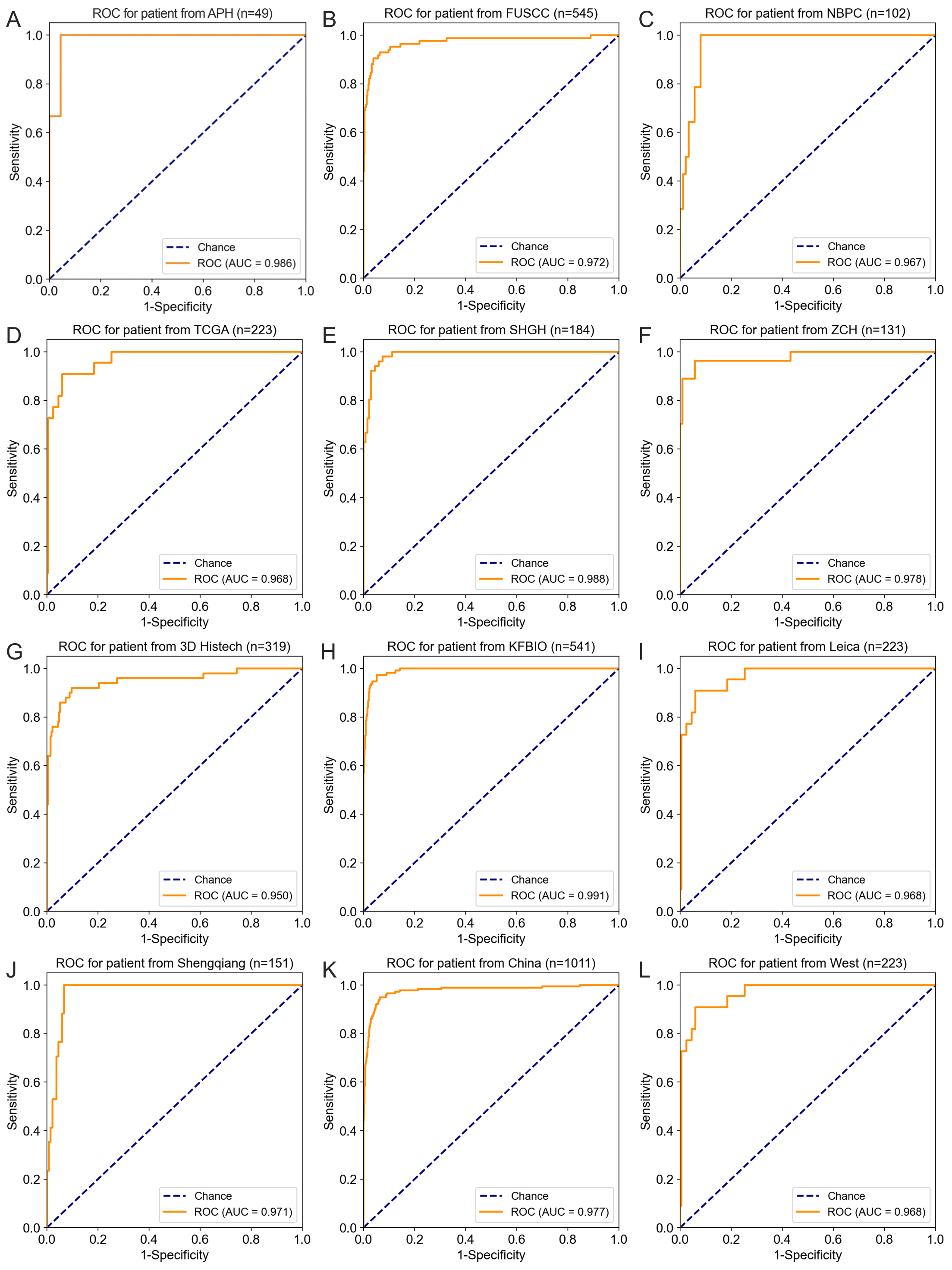

Figure S2. Confusion matrix of the Deepath-MSI model in (A-F) centers, (G-J) scanners, and (K-L) race.

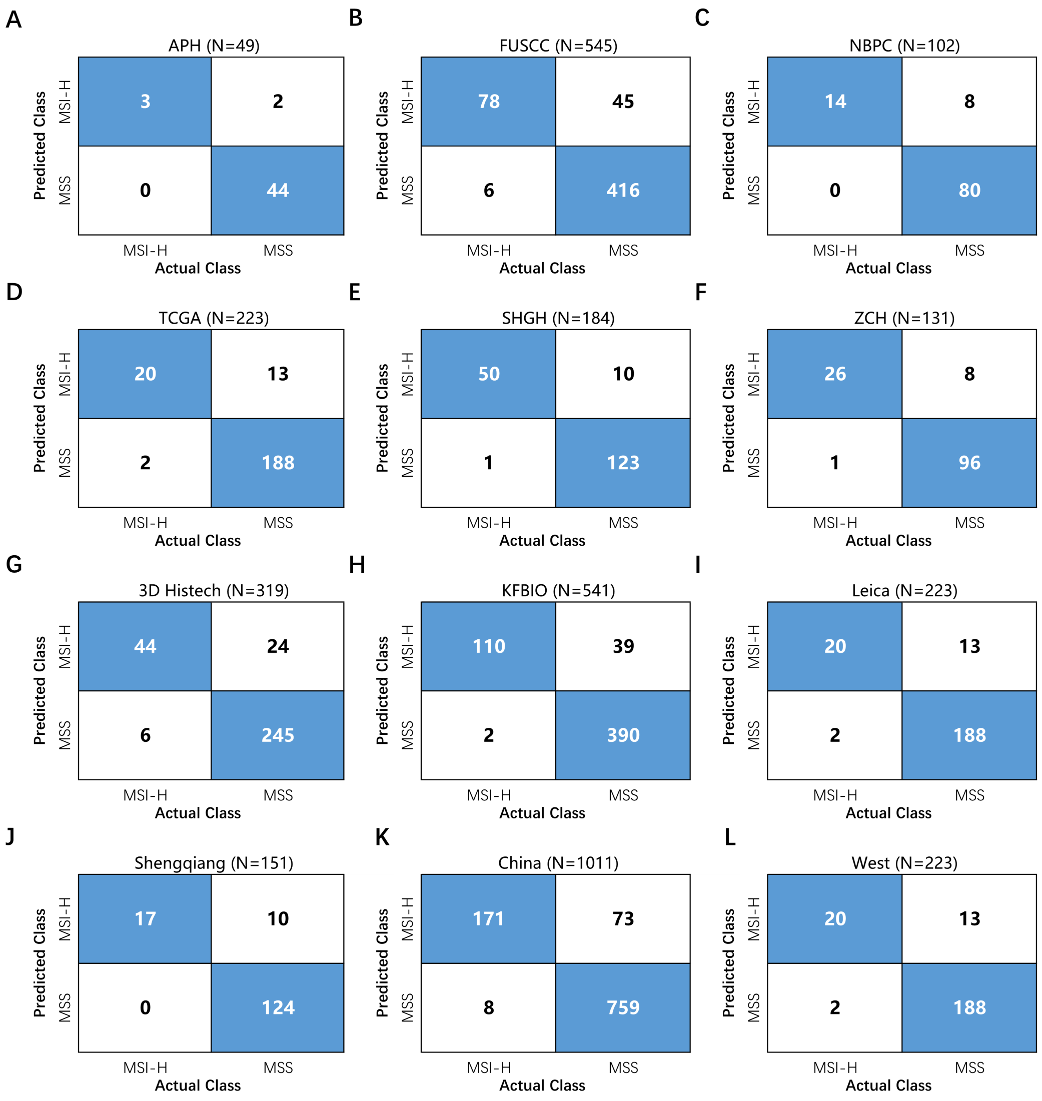

Supplement: Supplementary file 1 — Supplementary material [file 41698_2025_1094_MOESM1_ESM.pdf]
